# Early lens extraction with intraocular lens implantation for the treatment of primary angle closure glaucoma: an economic evaluation based on data from the EAGLE trial

**DOI:** 10.1136/bmjopen-2016-013254

**Published:** 2017-01-13

**Authors:** Mehdi Javanbakht, Augusto Azuara-Blanco, Jennifer M Burr, Craig Ramsay, David Cooper, Claire Cochran, John Norrie, Graham Scotland

**Affiliations:** 1 Health Economics Research Unit, University of Aberdeen, Aberdeen, UK; 2 Wellcome-Wolfson Institute for Experimental Medicine, Queen's University Belfast, Belfast, UK; 3 School of Medicine, University of St Andrews, St Andrews, UK; 4 Health Services Research Unit, University of Aberdeen, Aberdeen, UK

**Keywords:** Cost-effectiveness, Lens extraction, Laser peripheral iridotomy, randomised controlled trial, Angle closure glaucoma, QALY

## Abstract

**Objective:**

To investigate the cost-effectiveness of early lens extraction with intraocular lens implantation for the treatment of primary angle closure glaucoma (PACG) compared to standard care.

**Design:**

Cost-effectiveness analysis alongside a multicentre pragmatic two-arm randomised controlled trial. Patients were followed-up for 36 months, and data on health service usage and health state utility were collected and analysed within the trial time horizon. A Markov model was developed to extrapolate the results over a 5-year and 10-year time horizon.

**Setting:**

22 hospital eye services in the UK.

**Population:**

Males and females aged 50 years or over with newly diagnosed PACG or primary angle closure (PAC).

**Interventions:**

Lens extraction compared to standard care (ie, laser iridotomy followed by medical therapy and glaucoma surgery).

**Outcome measures:**

Costs of primary and secondary healthcare usage (UK NHS perspective), quality-adjusted life years (QALYs) and the incremental cost-effectiveness ratio (ICER) for lens extraction versus standard care.

**Results:**

The mean age of participants was 67.5 (8.42), 57.5% were women, 44.6% had both eyes eligible, 1.4% were of Asian ethnicity and 35.4% had PAC. The mean health service costs were higher in patients randomised to lens extraction: £2467 vs £1486. The mean adjusted QALYs were also higher with early lens extraction: 2.602 vs 2.533. The ICER for lens extraction versus standard care was £14 284 per QALY gained at three years. Modelling suggests that the ICER may drop to £7090 per QALY gained by 5 years and that lens extraction may be cost saving by 10 years. Our results are generally robust to changes in the key input parameters and assumptions.

**Conclusions:**

We find that lens extraction has a 67–89% chance of being cost-effective at 3 years and that it may be cost saving by 10 years.

**Trial registration number:**

ISRCTN44464607; Results.

**Strengths and limitations of this study**

- The analysis is based on randomised data collected prospectively as part of a pragmatic randomised controlled trial which included 285 participants recruited from 22 healthcare centres across the UK.
- Adequate randomisation and intention to treat analysis are further strengths of this study, which enhance the internal and external validity of our findings.
- Estimates of cost-effectiveness beyond three years rely on extrapolation of the trial data.
- As insufficient details were collected to allow for bottom-up costing of all relevant procedures, Healthcare Resource Group-based reference costs were used.

## Introduction

Glaucoma has been ranked as the second most common cause of blindness worldwide after cataract and is the leading cause of irreversible blindness.^1^ ^2^ A recent study estimated a global prevalence of 3.54% in people aged 40–80 years, with 64.3 million individuals estimated to be living with the condition in 2013. This number is projected to increase to 76.0 and 111.8 million by 2020 and 2040, respectively.^1^ There are two main types of glaucoma: open angle and angle closure. Although primary open angle glaucoma (POAG) is more prevalent, primary angle closure glaucoma (PACG) is more severe and more likely to result in irreversible blindness. In PACG, the drainage pathway at the anterior chamber angle of the eye is closed leading to increased intraocular pressure (IOP) which damages the optic nerve causing vision loss. In an earlier form of the disease, primary angle closure (PAC), there is increased IOP and an elevated risk of progression but no evident damage to the optic nerve. PACG is most prevalent in East Asia. The prevalence of PACG in the UK adult population (≥40 years old) is 0.4% (ie, there are about 130 000 patients in the country).^3^ Demographic risk factors are Chinese race, female gender and age. Blindness places a high economic burden on individuals, health systems and society as a whole,^4^ and the effect of severe glaucoma on quality of life is also profound.^5^

Current treatment for PACG follows a staged approach involving a combination of laser and medical management followed by glaucoma surgery.^6^ Irrespective of disease stage at diagnosis, laser iridotomy is the primary treatment procedure and eye drops are often required as an adjunct to further reduce the IOP. If treatments do not sufficiently reduce the IOP, then glaucoma surgery (eg, trabeculectomy) is indicated. However, glaucoma surgery may fail to control the condition and complications are more likely than for other types of glaucoma. The standard approaches to PACG management have been noted to have variable success.^7^ ^8^

Since the lens of the eye plays an important role in the development of PACG, it has been hypothesised that early lens extraction by phacoemulsification may improve control of IOP and thus reduce the need for medications and subsequent glaucoma surgery.^9^ By replacing laser iridotomy in the care pathway, lens extraction may enable patients to maintain better visual function and health-related quality of life.^9^ Following lens extraction, either trabeculectomy and/or use of a glaucoma drainage device offer follow-up surgical options for medically uncontrolled glaucoma.

The evidence for the effectiveness and cost-effectiveness of lens extraction compared to other treatment options for PACG is sparse. Friedman and Vedula^10^ conducted a systematic review to assess the effectiveness of lens extraction for chronic angle closure glaucoma compared with other interventions. They found two non-randomised studies of poor quality, which provided insufficient evidence to assess efficacy in terms of IOP control. The EAGLE trial was conducted to assess the clinical and cost-effectiveness of early lens extraction compared with standard care in individuals with PAC or PACG (ISRCTN 44464607). Here, we report on the results of the economic evaluation.

## Methods

### Study design

Details of the trial design have been reported in the study protocol.^9^ In brief, a parallel group randomised controlled trial (RCT) was conducted. Patients were recruited from 30 centres across the UK and 6 other countries—Malaysia, Singapore, Australia, Taiwan, Hong Kong and China. In total, 419 individuals were recruited and randomised (1:1) to early lens extraction (n=208) or standard care (n=211). The economic analysis adopts a UK health and social care perspective and is therefore based on the data from 285 participants recruited from 22 centres across the UK, with 145 randomised to lens extraction and 140 randomised to standard care between June 2009 and August 2012.

All eligible patients were identified by an ophthalmologist during their initial consultation. Individuals aged 50 years or over with newly diagnosed PAC and IOP ≥30 mm Hg, or PACG either untreated or under medical treatment for 6 months or less, were considered eligible. Exclusion criteria included: advanced glaucoma (determined by either: (1) visual field loss (mean deviation (MD) worse than −15 dB) or (2) cup–disc-ratio ≥0.9), previously diagnosed acute angle closure attack in an otherwise eligible eye, increased surgical risk (eg, corneal opacity, Fuch's endothelial dystrophy; pseudoexfoliation, previous vitreoretinal surgery, not able to be positioned to undergo standard technique), symptomatic cataract in either eye (defined as sufficient lens opacity such that one would normally recommend cataract surgery to relieve visual symptoms), prior cataract surgery or laser iridotomy in the study eye, axial length <19 mm (nanophthalmos), secondary angle closure glaucoma, retinal ischaemia, macular oedema, wet age-related macular degeneration (AMD) or being medically unfit for surgery or completion of the trial. Random allocation of the patients was performed using a web-based randomisation application with a minimisation algorithm that included gender, ethnicity, centre, diagnosis (PAC or PACG), and one or both eyes eligible. At each site, patients in the treatment group underwent phacoemulsification and intraocular lens implant within 60 days of randomisation, and those who were randomised to standard care were managed with laser peripheral iridotomy (standard practice). For patients with both eyes eligible, the worst eye (or the patient's choice if both eyes were equally affected) was designated the index eye and underwent treatment first. It was specified that second eligible eyes should receive the same intervention as the index eye within 60 days. Other subsequent treatments in both eyes (eg, medical therapy, laser peripheral iridoplasty and glaucoma surgery) were recorded up to 36 months postrandomisation. Patients had associated medical treatment (with eye drops) as needed to control the IOP, but if the disease was uncontrolled the patient underwent glaucoma surgery. The type of glaucoma surgery was chosen by the surgeon. Those randomised to standard care could undergo lens extraction during the study period only when indicated clinically for reduced vision (ie, cataract surgery), or if the treating physician felt lens extraction could help control the IOP after escalation to maximum medical treatment had failed (ie, glaucoma surgery). The primary economic outcome was the incremental cost per quality-adjusted life year (QALY) gained, with QALYs assessed using the EQ-5D 3 level.^11^

### Health resource use and costs

Secondary care resource use was collected on case report forms (CRF). Use of primary care services was collected from patient questionnaires delivered at baseline, 6, 12, 24 and 36 months. The costs of surgical or non-surgical procedures were estimated based on data recorded in the trial CRF combined with national unit cost data for each specific procedure (table 1).^12^ Primary care usage, including general practitioner (GP) contacts, district/practice nurse consultations and community optician/optometrist visits, was valued using published per visit unit costs.^13^ The type and dose of medications administered to patients was collected at each follow-up time point. Total medication costs were estimated based on the type and duration of medical treatment, combined with the associated unit costs.^14^ Finally, all cost elements of the interventions and subsequent health service use were summed to generate a total cost per patient. All unit costs were obtained for the financial year 2012–2013.

**Table 1:** Main unit costs applied in the analysis

Input variables	Unit cost (£)	Source	Details
Interventions			
Lens extraction implemented as day case	866	National schedule of reference costs year 2012–2013	Phacoemulsification cataract extraction and lens implant implemented as day case (BZ02)
Lens extraction implemented as inpatient	2157	National schedule of reference costs year 2012–2013	Phacoemulsification cataract extraction and lens implant implemented as inpatient (BZ02)
Laser iridotomy implemented as outpatient	118	National schedule of reference costs year 2012–2013	Laser iridotomy (minor glaucoma procedures implemented as outpatient (BZ19))
Subsequent procedures			
Lens capsulotomy	121	National schedule of reference costs year 2012–2013	Lens capsulotomy (BZ04) implemented as outpatient
Iridoplasty	172	National schedule of reference costs year 2012–2013	Major glaucoma procedures implemented as outpatient (BZ17)
Trabeculectomy	1140	National schedule of reference costs year 2012–2013	Intermediate glaucoma procedures implemented as day case (BZ18)
Cataract surgery	866	National schedule of reference costs year 2012–2013	Phacoemulsification cataract extraction and lens implant implemented as day case (BZ02)
Primary healthcare			
General practitioner visit	43	PSSRU 2013	Community-based healthcare staff
General practitioner visit at home	53.58	PSSRU 2013	Community-based healthcare staff
General practitioner telephone conversation	26	PSSRU 2013	Community-based healthcare staff
Community optician and optometrist	62	National schedule of reference costs year 2012–2013	Follow-up attendance—non-consultant led outpatient attendances
District nurse	12.40	PSSRU 2013	Community-based healthcare staff
Practice nurse	10.59	PSSRU 2013	Community-based healthcare staff
Clinical support worker nursing (community)	31	PSSRU 2013	Community-based healthcare staff
Secondary healthcare			
Ophthalmologist visit	80	National schedule of reference costs year 2012–2013	Consultant-led outpatient attendances, follow-up

### Costs incurred by participants and indirect costs

Participant costs were estimated from responses to the follow-up questionnaires to 36 months and included self-purchased healthcare and travel costs associated with making return visit(s) to NHS healthcare facilities. Self-purchased healthcare costs included items such as prescription costs, over the counter medications and costs associated with spectacle wear. These were calculated based on the amounts that patients reported paying for them. Patient travel costs were calculated based on patient reported modes of travel and associated costs, multiplied by the number of visits to each type of facility. Indirect costs, encompassing time costs for accessing NHS healthcare and time lost from productive activities due to ill health, were estimated based on the reported times taken to attend appointments and reported time away from usual activities (eg, paid work, leisure time, housework). Data on wage rates were taken from the Department of Work and Pensions and used to value time lost from paid or unpaid employment.^15^ Inferred values for housework and leisure time were obtained from other published sources.^16^ ^17^

### Effectiveness

Effectiveness was measured in terms of QALYs gained. QALYs were estimated based on participant responses to the EQ-5D completed at baseline, 6, 12, 24 and 36 months. Reported health status at each time point was assigned a utility score using the UK population time trade-off tariff.^18^ The QALYs for each participant were calculated by multiplying the time spent in different states of health by the utility score associated with each state, assuming a linear change in utility between time points. A zero utility weight was assigned from the time of death for those participants who died during study follow-up. The Glaucoma Utility Index (GUI) was also administered as an alternative disease-specific preference-based measure of health-related quality of life.^19^ The GUI dimensions include central and near vision; lighting and glare; activities of daily living; mobility; eye discomfort; and other effects. This instrument has been scored using a discrete choice experiment conducted on a sample of individuals with glaucoma, providing a preference-based index value on a scale where 0 is equal to the worst state and 1 is equal to the best state described by the instrument.

### Statistical analysis of trial data

All data were analysed on an intention-to-treat basis using Stata V.12 (StataCorp. 2011. Stata Statistical Software: Release 12. College Station TSL). Healthcare cost and utility data often have several characteristics that must be addressed through the careful selection of appropriate statistical analysis methods. In this study, different regression models including generalised linear models (GLM) with appropriate variance and link functions, seemingly unrelated regression (SUR) and ordinary least square (OLS) were used to estimate the effect of treatment allocation on costs and QALYs after adjusting for minimisation factors and appropriate prognostic covariates at baseline (ie, baseline cost and EQ-5D score). SUR was used for the primary analysis to estimate between-group differences in mean costs and QALYs, while accounting for correlation in the error terms. The method of recycled predictions was used to estimate the incremental effect of the treatment indicator variable.^20^ All analyses were also repeated using a multiple imputation (MI) data set (n=20) which was generated using chained equations to deal with missing cost and utility data (StataCorp. Stata Multiple-Imputation Reference Manual Release 12. StataCorp LP: College Station, 2012).

The estimates of mean costs and effects of the two strategies were compared in an incremental analysis, to estimate the incremental cost-effectiveness ratio (ICER) for lens extraction versus standard care. The ICER is calculated as the difference in costs divided by the difference in effects (QALYs) between two treatments. The uncertainty surrounding the joint incremental costs and effects was presented graphically as confidence ellipses on the incremental cost-effectiveness plane. Since the ICER has poor statistical properties where differences in effects are very small, we used the net monetary benefit (NMB) framework to ascertain the probability of each strategy being cost-effective at different ceiling ratios (Rc) representing decision makers' maximum willingness to pay per QALY gained. The NMB for a given strategy is equal to the mean QALYs accrued multiplied by Rc, minus the strategy costs.1
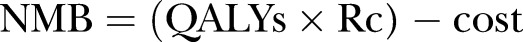


By generating 1000 bootstrapped replicates of the mean difference in costs and effects, the proportion of replicates favouring each strategy (in terms of mean NMB) was calculated for a range of plausible values of Rc. These proportions are interpreted as probabilities of each strategy being cost-effective at 3 years for the different values of Rc. On the basis of the NICE guidance,^21^ we report these probabilities at ceiling ratios of £20 000 and £30 000 per QALY gained. The mean incremental NMB (95% CIs) for lens extraction versus standard care was also plotted against increasing values of Rc. Within trial subgroup analysis was also conducted to assess how the estimated ICER varied by disease status (PAC vs PACG) and one or both eyes eligible for the study.

### Markov model for extrapolation of longer term cost-effectiveness

**Figure 1:**
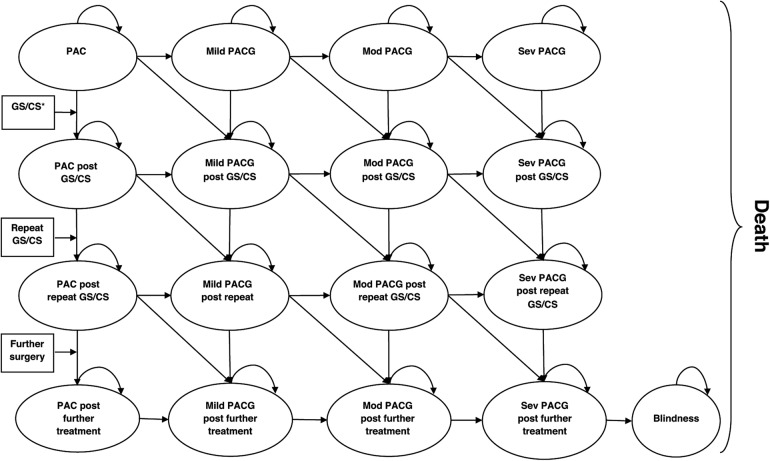
Structure of the Markov model. PAC, primary angle closure; PACG, primary angle closure glaucoma.

Although the within trial results provide useful information about the cost-effectiveness of lens extraction versus standard care, the effects of treatment on cumulative costs and QALYs are expected to persist further into the future. Therefore, a Markov model was developed to extrapolate the results of the trial beyond the 3-year follow-up period to 5 and 10 years (figure 1). The model was developed Using TreeAge Pro 2014 (TreeAge Pro 2014, R1.0. TreeAge Software, Williamstown, MA; software. http://www.treeage.com) to simultaneously capture disease progression through glaucoma severity states and progression to subsequent surgical treatment (figure 1). The glaucoma severity states were defined using cut-offs on the Enhanced Glaucoma Staging System (GSS 2), which is calculated from two visual field measures: the MD and the pattern SD (PSD).^22^ The GSS 2 categorises visual field damage on a scale from 0 to 16. We used the modified visual field staging system proposed by Che Hamzah *et al*^23^ to create the following glaucoma severity stages: PAC/normal (0), mild (1–4), moderate (5–10) and severe (11–16). The probability of progression was defined as moving down one stage or more by 36 months. These probabilities were estimated by baseline severity level and treatment allocation group using logistic regression on the trial data and transformed into constant 6 month probabilities for use in the Markov model.

The model structure allows a newly diagnosed cohort of patients with varying degrees of glaucoma severity to enter the model and then follow the treatment sequence described above. The model is updated iteratively on a constant 6-month time interval known as the Markov cycle. The mean age and sex distribution of the modelled cohort matched that of the trial participants at baseline. During each model cycle, a portion of the cohort progresses in severity (from PAC to mild, mild to moderate or moderate to severe) based on the probabilities of progression derived from the analysis of the trial data. Within each model cycle, a proportion of the cohort also transits to glaucoma or cataract surgery (ie, trabeculectomy, minimally invasive glaucoma surgeries (MIGS), Ahmed tube, Zonulo-hyaloido-vitrectomy, clear lens extraction to control IOP or cataract surgery). This is based on time-dependent transition probabilities derived from a Weibull regression of the observed time to surgery up to 36 months follow-up (see online supplementary table S1). The Weibull distribution was selected over other potential candidate distributions based on the Bayesian information criterion. However, we also assessed the impact of modelling progression to surgery using an exponential distribution. Finally, death from all causes is included in the model as an absorbing state. Transition probabilities to this state are assumed to be independent of glaucoma severity and treatment history and are derived from age/sex specific UK life tables.^24^

10.1136/bmjopen-2016-013254.supp1 supplementary tables and figures

Costs are assigned to each state in the model, reflecting the mean monitoring and medication costs per 6-month cycle by glaucoma severity and treatment allocation. To populate the model, we disaggregated the total monitoring and medication costs incurred within the trial follow-up period to those incurred between each follow-up time point. This was carried out to best reflect the trend in health services usage over time following initial intervention. Costs associated with progression to glaucoma or cataract surgery were incorporated as transition costs for those modelled to experience these events. Utility values were also attached to the modelled severity states by the treatment allocation group, allowing cumulative model-based QALYs to be estimated.

Beyond 36 months in the model, we assumed that the mean cost and utility values (by clinical severity state and treatment allocation) would be the same as those incurred between 30 and 36 months. Those modelled to transit to cataract or glaucoma surgery over follow-up in the standard care group were modelled to incur the same health state utility (by severity state) as observed for those randomised to early lens extraction from that point onwards in the model.

All model input parameters were defined as statistical distributions in the model, allowing probabilistic analysis to be conducted. Ranges and distributional assumptions for input parameters were based on the trial data and the literature. We assigned gamma distributions for costs and β distributions for utility data. We also calculated correlations between the estimated coefficients for the variables included in the time-to-event and logistic regression analyses using Cholesky decomposition and assigned multinormal distributions to these parameters in the model to account uncertainty in the estimated transition probabilities. The analysis was conducted using second-order Monte Carlo simulation, whereby the model was analysed 1000 times with a value randomly drawn for each input parameter from its assigned distribution. By estimating the NMB for each strategy for each iteration of the probabilistic analysis, cost-effectiveness acceptability curves were generated. These present the probability of each strategy being cost-effective across plausible ranges of Rc. All future costs and QALYs were discounted using a discount rate of 3.5% per annum.^21^

### Sensitivity analysis

Deterministic sensitivity analysis was conducted to investigate the impact of varying key assumptions and/or parameter values used in the base case analysis. We explored the impact of the following changes/scenarios: (1) excluding costs of procedures and medications in non-eligible eyes; (2) basing the model input parameters on analysis of the multiple imputation data set; (3) including indirect and patient costs; (4) using alternative regression models to estimate the mean cost and utility parameters (GLM); (5) changing the mean age of the cohort from 67 to 50 years; (6) adopting different time horizons and (7) estimating time to glaucoma or cataract surgery using exponential survival regression.

## Results

### Within trial analysis

The mean age of participants was 67.5 (8.42), 57.5% were women, 44.6% had bilateral disease with both eyes eligible for the study, 1.4% were of Asian ethnicity and 35.4% had PAC rather than PACG. Among randomised patients with one eye eligible for the study, 65% had bilateral disease but had one eye not eligible according to the study exclusion criteria. Baseline characteristics were similar between groups (table 2). Of the 285 patients randomised in the UK, 179 (62.8%) had complete cost and utility data; 93 (64.14%) in the lens extraction arm and 86 (61.43%) in the standard care arm (see online supplementary figure 1). The remaining participants had missing elements of cost or EQ-5D data, precluding calculation of total costs or QALYs. A total of 143 (98.62%) and 139 (99.28) patients received their intended treatment in the lens extraction and standard care group, respectively. Healthcare resource use is summarised in online supplementary table S2 (supporting data) by the treatment allocation group. The mean NHS costs were higher in patients randomised to lens extraction; £2474 compared to £1480 (table 4). The mean EQ-5D and GUI utility scores, unadjusted QALYs and IOP and visual acuity scores are summarised in table 3. The mean estimated EQ-5D index at baseline and 36 months was 0.881 (0.850–0.912) and 0.857 (0.817–0.897) in the lens extraction group and 0.872 (0.840–0.905) and 0.845 (0.803–0.888) in the standard care group. The mean estimated QALYs for patients randomised to lens extraction were 2.602 (2.527–2.672) compared to 2.533 (2.447–2.608) in the standard care group after adjusting for covariates. The mean estimated GUI at baseline and 36 months was 0.877 (0.852–0.901) and 0.919 (0.901–0.936) for the patients in the lens extraction group and 0.874 (0.847–0.901) and 0.871 (0.845–0.896) for those in the standard care group.

**Table 2:** Baseline characteristics of participants

	Randomised to early lens extraction	Randomised to standard care
	N=145	N=140
Variables	n (%)	n (%)
Female	85 (58.62)	79 (56.43)
Both eyes eligible	63 (43.45)	64 (45.71)
Index eye is right	78 (53.79)	80 (57.14)
Chinese	3 (2.07)	1 (0.71)
Diagnosis in index eye		
PAC	55 (37.93)	46 (32.86)
PACG	89 (61.38)	94 (67.14)
Missing	1 (0.69)	0 (0)
	**Mean (SD)**	**Mean (SD)**
Age	67.8 (8.48)	67.2 (8.37)
IOP in index eye	29.0 (8.60)	29.7 (7.78)
Visual acuity in index eye	80.5 (11.17)	80.4 (10.84)
Visual acuity in both eyes	84.6 (8.92)	85.6 (7.56)
EQ-5D score	0.881 (0.19)	0.872 (0.19)
Glaucoma Utility Index	0.877 (0.15)	0.874 (0.15)

PAC, primary angle closure; PACG, primary angle closure glaucoma.

**Table 3:** Health service usage costs and health outcome measures (EQ-5D and Glaucoma Utility Index) by intention to treat

Variables	Randomised to early lens extraction		Randomised to standard care	
Healthcare cost	N	£ Mean (SD)	N	£ Mean (SD)
Initial intervention cost (lens extraction/standard care)				
Intervention in eligible eyes	145	1229 (658)	139	181 (77)
Cost of subsequent procedures in eligible eyes				
Lens capsulotomy	122	14 (52)	113	0 (0)
Laser iridotomy	122	1 (11)	113	20 (63)
Iridoplasty	122	0 (0)	113	21 (77)
Trabeculectomy	122	19 (145)	113	57 (275)
Cataract surgery	122	28 (155)	113	188 (469)
Other procedures	122	18 (113)	113	45 (276)
Medication cost	122	36 (61)	113	115 (116)
Cost of procedures in non-eligible eyes				
Lens capsulotomy	122	2 (15)	113	0 (0)
Laser iridotomy	122	11 (34)	113	67 (70)
Iridoplasty	122	0 (0)	113	3 (23)
Trabeculectomy	122	0 (0)	113	0 (0)
Lens extraction/cataract surgery	122	302 (441)	113	15 (115)
Medication cost	122	13 (31)	113	27 (49)
Primary care costs				
GP visits	108	83 (144)	103	117 (223)
Nurse visits	108	27 (58)	102	147 (1115)
Community optician/optometrist visits	108	117 (112)	103	121 (117)
Secondary care costs				
Ophthalmology outpatient visits	122	373 (438)	113	407 (390)
Total NHS costs	107	2441 (886)	103	1509 (1389)
Participant cost	105	433 (449)	101	438 (387)
Indirect cost	105	725 (1131)	101	532 (755)
	**N**	**Mean (SD)**	**N**	**Mean (SD)**
EQ-5D values				
Baseline	142	0.881 (0.189)	135	0.872 (0.190)
6 months	125	0.903 (0.185)	124	0.847 (0.233)
12 months	130	0.905 (0.159)	120	0.847 (0.232)
24 months	126	0.883 (0.196)	121	0.847 (0.241)
36 months	125	0.857 (0.228)	116	0.845 (0.229)
Total QALYs	96	2.585 (0 0.427)	88	2.526 (0.488)
GUI				
Baseline	142	0.877 (0.149)	133	0.874 (0.155)
6 months	126	0.919 (0.094)	127	0.880 (0.138)
12 months	132	0.912 (0.096)	122	0.879 (0.125)
24 months	123	0.916 (0.096)	120	0.878 (0.125)
36 months	128	0.919 (0.102)	122	0.871 (0.142)
Intraocular pressure				
Baseline	145	29.0 (8.6)	140	29.7 (7.8)
6 months	139	16.0 (3.9)	134	19.7 (5.3)
12 months	138	16.2 (3.3)	128	18.8 (4.4)
24 months	138	17.3 (4.2)	124	19.1 (5.0)
36 months	130	16.9 (3.8)	124	18.1 (3.8)
Visual acuity (ETDRS)				
Baseline	145	80.5 (11.2)	135	80.4 (10.8)
12 months	129	83.0 (7.1)	126	80.9 (11.1)
36 months	129	81.8 (8.4)	122	79.8 (12.2)

GUI, Glaucoma Utility Index; QALY, quality-adjusted life years.

**Table 4:** Incremental cost-effectiveness measures (within trial analysis)

							Probability cost-effective at Rc	
Data	Intervention	Cost (£)	ΔCost (£)	QALY	ΔQALY	ICER (ΔCost/ ΔQALY) (£)	£20 000	£30 000
Complete case*	Standard care	1486	981	2.533	0.069	14 284	0.671	0.776
	Lens extraction	2467		2.602				
Multiple imputation	Standard care	1567	844	2.442	0.100	8430	0.885	0.940
	Lens extraction	2411		2.542				

*179 of 285 (93 (64.14%) in the lens extraction and 86 (61.43%) in the standard care group) UK participants have complete cost and QALY data—above regression results are based on the data of these complete cases.

ICER, incremental cost-effectiveness ratio; QALY, quality-adjusted life years; Rc, ceiling ratio of willingness to pay per QALY gained.

**Figure 2:**
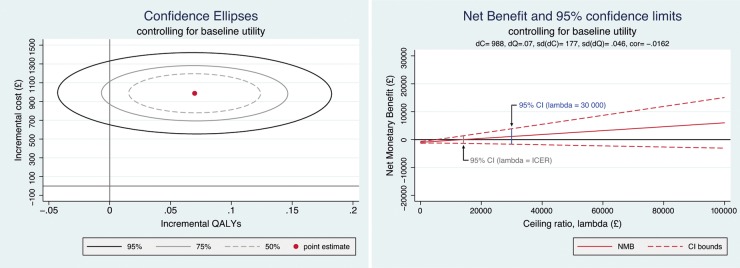
Confidence ellipses and NMB plot. NMB, net monetary benefit.

The analysis of complete case data gives an incremental cost estimate of £981 (612–1317) for lens extraction versus standard care, for a mean QALY gain of 0.069 (−0.017–0.159), yielding an ICER of £14 284 per QALY gained (table 4). On the basis of 1000 bootstrapped estimates of the differences in the mean cost and effects, lens extraction has a 67% probability of being cost-effective at 3 years; that is, a 67% probability of generating the greatest NMB at a ceiling willingness-to-pay ratio (Rc) of £20 000 per QALY. This increases to 78% for an Rc of £30 000 per QALY. Confidence ellipses (50%, 75% and 95%) for the joint differences in costs and effects and the NMB plot for the complete case analysis are presented in figure 2.

Considering patient and indirect costs, the mean estimates were £430 (332–553) and £685 (495–933), respectively, for patients randomised to lens extraction, compared to £479 (372–576) and £489 (335–670) for patients randomised to standard care.

The analysis based on multiple imputation for missing data suggests that patients randomised to lens extraction are expected to cost the health service £844 (551–1124) more on average for a mean QALY gain of 0.1 (0.016–0.193). The estimated ICER is £8430 per QALY gained, and the probability of lens extraction being cost-effective is 88.5% at an Rc of £20 000 per QALY.

Finally, the incremental cost-effectiveness results are presented by the diagnosis group and by one or both eyes eligible in table 5. The mean incremental costs and QALYs for lens extraction versus standard care in patients with PAC were £1046 (611–1513) and 0.078 (−0.057–0.203), respectively, whereas the corresponding figures were £963 (468–1421) and 0.067 (−0.040–0.190) among patients with PACG. The estimated ICERs in patients with PAC and PACG were £13 401 and £14 462 per QALY gained, respectively. The mean incremental costs and QALYs in patients with one eye eligible were £915 (£348–£1344) and 0.004 (−0.105–0.109), respectively, with a corresponding ICER of £209 173 per QALY gained. For those with both eyes eligible the incremental cost was £1099 (£540–£1631) for an estimated QALY gain of 0.162 (−0.005–0.329), yielding an ICER of £6765 per QALY gained.

**Table 5:** Incremental cost-effectiveness measures by diagnosis subgroups based on complete case* data (within trial analysis)

							Probability cost-effective at Rc	
Subgroup	Intervention	Cost (£)	ΔCost (£)	QALY	ΔQALY	ICER (ΔCost/ΔQALY) (£)	£20 000	£30 000
PAC	Standard care	1374	1046	2.553	0.078	13 401	0.661	0.742
	Lens extraction	2420		2.631				
PACG	Standard care	1535	963	2.522	0.067	14 462	0.620	0.713
	Lens extraction	2497		2.589				
One eye eligible	Standard care	1397	915	2.595	0.004	209 173	0.244	0.317
	Lens extraction	2312		2.600				
Both eyes eligible	Standard care	1595	1099	2.450	0.162	6765	0.901	0.938
	Lens extraction	2695		2.613				

*179 of 285 (93 (64.14%) in the lens extraction and 86 (61.43%) in the standard care group) UK participants have complete cost and QALY data—above regression results are based on the data of these complete cases.

ICER, incremental cost-effectiveness ratio; QALY, quality-adjusted life years; Rc, ceiling ratio of willingness to pay per QALY gained.

### Model-based analysis

**Figure 3:**
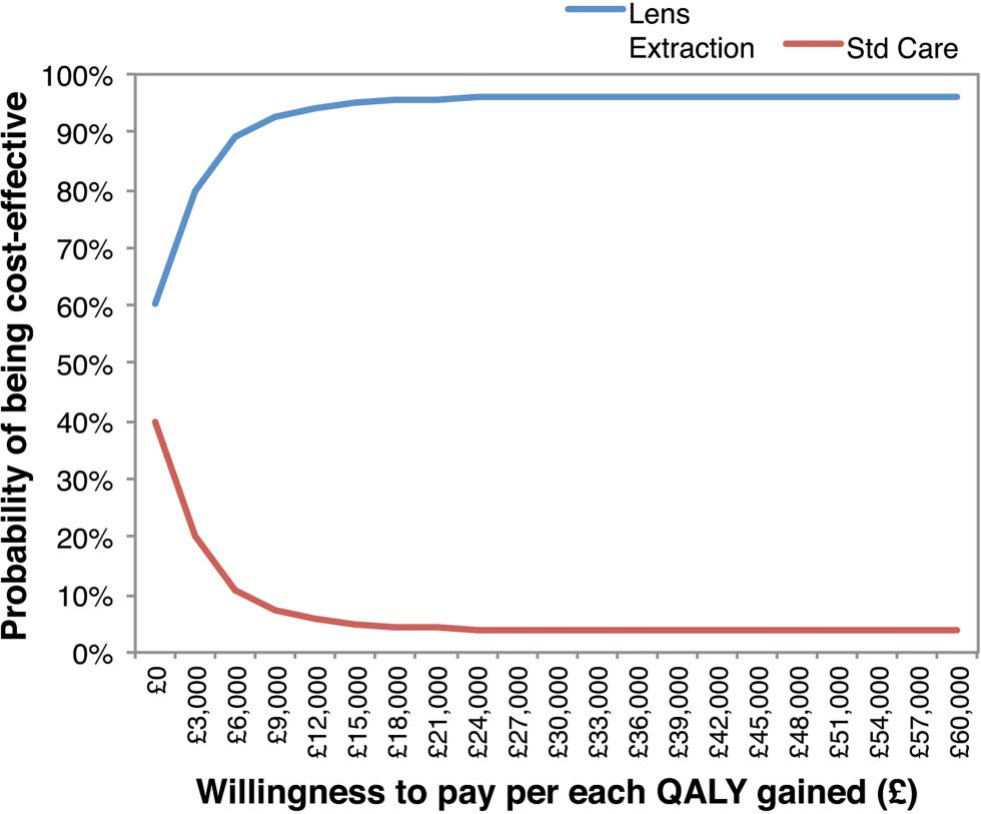
Model-based cost-effectiveness acceptability curve for a 10-year time horizon.

The model-based estimates of mean costs and QALYs at 3-years indicate that lens extraction is expected to cost an additional £938 on average for a QALY gain of 0.062 vs standard care, with a corresponding ICER of £15 223 per QALY gained (table 6). The projected incremental cost and QALY gain associated with lens extraction at 5 years is estimated to be £559 and 0.079, respectively (ICER=£7090). Running the model over a 10-year time horizon suggests that lens extraction may dominate standard care by this time point; that is, result in a lower net cost to the health system while generating more QALYs. The model-based probabilities of lens extraction being cost-effective at 3, 5 and 10 years are 0.695, 0.897 and 0.957, respectively (table 6). Figure 3 presents the model-based cost-effectiveness acceptability curves based on the 10-year time horizon.

**Table 6:** Incremental cost-effectiveness measures (model-based analysis)

							Probability cost-effective at Rc	
Time horizon	Intervention	Cost (£)	ΔCost (£)	QALY	ΔQALY	ICER (ΔCost/ ΔQALY) (£)	£20 000	£30 000
3-years' time horizon	Standard care	1563	938	2.484	0.062	15 223	0.699	0.887
	Lens extraction	2501		2.546				
5-years’ time horizon	Standard care	2264	559	3.903	0.079	7090	0.897	0.951
	Lens extraction	2823		3.982				
10-years’ time horizon	Standard care	3481	−123	6.620	0.107	cost saving	0.957	0.961
	Lens extraction	3358		6.727				

ICER, incremental cost-effectiveness ratio; QALY, quality-adjusted life years; Rc, ceiling ratio of willingness to pay per QALY gained.

### Sensitivity analysis

Deterministic sensitivity analysis shows that the model-based findings are generally robust to the changes examined. Excluding the costs of procedures and medications in non-eligible eyes reduced the estimated ICER from £15 223 to £11 721 at 3 years. Using the multiple imputation data set to parameterise the model, the estimated ICER for lens extraction dropped to £8267 at 3 years. The estimated ICER based on a 3-year time horizon varied from £13 952 to £16 772 for the other remaining scenarios assessed. Results of all other scenario analyses are presented in online supplementary table S3.

## Discussion

### Principal findings

Our results suggest that lens extraction is likely to offer a cost-effective approach to treatment compared with standard care in patients with PACG or PAC, especially over the longer term. Although the initial costs are higher for patients assigned to lens extraction, these are partly offset by cost savings associated with fewer subsequent procedures (including repeat laser iridotomy, lens extraction or glaucoma surgery) and lower medication use over 3 years. On the basis of the Markov modelling, our results suggest that lens extraction may become a cost-saving strategy over a 10-year time horizon.

It should be noted that there is a small difference in the estimated mean costs and QALYs between the within trial and model-based analyses at 3 years. These differences are explained partly by the way we used the trial data to populate our model and also random noise in the Monte Carlo simulation. A further difference between the trial and model-based analysis is that the model references age-specific and sex-specific probabilities of death derived from UK life tables, rather than the observed within trial mortality. This also partly explains small differences in mean costs and QALYs.

Within trial subgroup analysis indicated that the estimated ICERs are very similar for those with PAC and PACG. The point estimate of the ICER for patients with only one eye eligible was considerably higher compared to the base case ICER. This is driven by a smaller QALY gain in this subgroup compared to those with two eyes eligible. However, it should be noted that the interaction between treatment allocation and QALYs was not significant at the traditional 5% type one error level, and so this may be a chance finding due to small numbers. In addition, it should be noted that 65% of ineligible eyes had established PAC/PACG and were considered ineligible based on the study exclusion criteria (ie, advanced glaucoma, previously diagnosed acute angle closure attack, any previous intraocular procedure or laser treatment). The existing pathology in the ineligible eyes of these patients may have dominated their HRQoL and limited capacity to detect improvements associated with treatment to the eligible eye. Thus, the results in this subgroup are not applicable to patients newly diagnosed with unilateral disease where the other eye is unaffected. Such patients would likely go on to develop bilateral disease (∼90% of eyes of participants in this study were diagnosed with either PAC or PACG) and so could have both eyes treated with early lens extraction at the appropriate time. Thus, it is not unreasonable to assume that all patients presenting with newly diagnosed angle closure glaucoma (unilateral or bilateral) could ultimately see QALY gains in line with those observed for patients with both eyes eligible in EAGLE.

### Strengths and limitations

Health service resource use and utility data were collected prospectively as part of this pragmatic RCT which included 285 participants recruited from 22 healthcare centres across the UK. The results of this economic analysis should therefore be generalisable across the UK NHS for patients with early or moderate PACG, and those with PAC and very high IOP (of 30 mm Hg or more). Furthermore, data were collected on procedures undertaken in both eyes of eligible patients, which should ensure that the estimated change in costs with lens extraction versus standard care is reflective of what would be expected if such a policy were adopted at a national level. Adequate randomisation and intention to treat analysis are further strengths of this study, which enhance the internal and external validity of our findings. In addition, conducting a parallel model-based analysis and populating it with data derived from the trial is another strength. This provided the flexibility to extrapolate beyond the trial follow-up period for the purpose of better informing decision-making. Finally, we conducted sensitivity analysis to provide information on how the cost-effectiveness of lens extraction may vary with changes to key parameters and assumptions.

Nevertheless, our study has some limitations that need to be considered when interpreting the results. First, as insufficient details were collected to allow for bottom-up costing of all relevant procedures, Healthcare Resource Group (HRG) based reference costs were used. As each unit cost in the HRG reference costs is for a group of procedures with slightly different average costs, there is a chance these could over or underestimate the real cost of each procedure. However, the applied reference costs were generally specific to the glaucoma procedures of relevance in the EAGLE trial and were also applied by treatment setting (inpatient, day case, and outpatient) to maximise precision. Furthermore, the fact that these reference costs are based on national routine data may improve generalisability.

Since trial-based economic evaluation requires complete cost and health-related quality of life data across all follow-up time points, this precluded 37% of patients being included in the complete case analysis. To address uncertainty arising from these missing data, we used multiple imputation based on chained equations to fill in numerous plausible values for missing cost and utility elements. This assumes that data are ‘missing at random’; that is, missingness can be explained by observed outcomes and variables. It is a more realistic assumption in many ways than the assumption of complete case analysis, which is that data are ‘missing completely at random’ (ie, as if a random sample of data points has been removed). The results of the multiple imputation (MI) suggest that the complete case analysis may underestimate the difference in effects between the alternatives, as a result of those with poorer health outcomes being less likely to respond to questionnaires (particularly affecting the standard care group).

To extrapolate the results beyond the trial follow-up period, we applied constant mean severity state costs reflecting costs of medications and monitoring in year 3 of the trial. However, this is justified by the fact that these costs were observed to be fairly constant over the second and third year of follow-up. In addition, larger resource use events (subsequent glaucoma or cataract surgery) were modelled explicitly based on time-dependent transition probabilities derived from parametric survival analysis. This should improve the precision of cost projections beyond three years in the model. Finally, due to data collection limitations in the trial we were not able to account for pharmacist time in our cost estimations. However, given that the usage of medications was higher among the standard care group, including these costs would further decrease the ICER in favour of early lens extraction.

### Comparison with other studies

Owing to the paucity of studies on the effectiveness and cost-effectiveness of early lens extraction as an initial treatment for patients with PACG, it is difficult to compare our results with other studies. Patients with acute attacks of angle closure (a rare presentation of the disease) were excluded in our trial, and thus studies evaluating lens extraction for acute attacks are not comparable.^25^ ^26^ A review conducted by Trikha *et al*^8^ has suggested that lens extraction for PACG is associated with better IOP control, reduced complication rates and reduced need for IOP-controlling medications compared to other relevant treatments. These findings are in keeping with ours.

### Implications and conclusions

The present study indicates that lens extraction has a 67–89% chance of being cost-effective at 3 years (assuming willingness to pay value of £20 000 per QALY gained), and on the basis of extrapolation it may be cost saving by 10 years. Early lens extraction appears likely to offer a cost-effective approach to treatment in patients with newly diagnosed PAC or PACG. Further randomised studies would help to confirm these findings, and longer term follow-up of patients enrolled in this study would help to verify the model-based extrapolations reported here.
