# Supplementary material for: Early lens extraction with intraocular lens implantation for the treatment of primary angle closure glaucoma: an economic evaluation based on data from the EAGLE trial
Source: BMJ Open. 2017 Jan 13;7(1):e013254. doi: 10.1136/bmjopen-2016-013254 (PMC5253715; doi:10.1136/bmjopen-2016-013254)
Supplement: supplementary tables and figures [file bmjopen-2016-013254supp_figures_and_tables.pdf]

Supplementary Figure 1: Consort diagram for UK trial participants

Assessed for eligibility N=554

Does not meet inclusion criteria	n=165(29.8%)
PAC with low IOP or angle closure<180 degrees or age<50	67(24.9%)
diagnosed more 6 months ago or previous iridotomy	40(14.9%)
other ocular co-morbidity	26(9.7%)
miscellaneous	16(5.9%)
severe PACG	7(2.6%)
medically unfit	4(1.5%)
open-angle glaucoma	3(1.1%)
secondary glaucoma (ACG)	2(0.7%)

Declined to participate n=104(18.8%)

PAC=33 PACG=28 PAC_L PACG_R=11 PAC_R PACG_L=9 missing=23

Randomised n=285(51.4%)

Lens extraction n=145		Standard care n=140	
PAC n=55	PACG n=89	PAC n=46	PACG n=94

Lens extraction		Standard care	
**6 months**		**6 months**	
Questionnaire completed	131(90.3%)	Questionnaire completed	129(92.1%)
No response	14(9.7%)	No response	8(5.7%)
eq5d reading	125(86.2%)	eq5d reading	124(88.6%)
iop reading	139(95.9%)	iop reading	134(95.7%)
Complete cost data	131 (90.3%)	Complete cost date	128 (91.4%)
withdrawal from study		withdrawal from study	3(2.1%)
**12 months**		**12 months**	
Questionnaire completed	133(91.7%)	Questionnaire completed	125(89.3%)
No response	11(7.6%)	No response	11(7.9%)
eq5d reading	130(89.7%)	eq5d reading	120(85.7%)
iop reading	138(95.2%)	iop reading	128(91.4%)
Complete cost data	132 (91.0%)	Complete cost data	122 (87.1%)
withdrawal from study	1(0.7%)	withdrawal from study	3(2.1%)
deceased		deceased	1(0.7%)
**24 months**		**24 months**	
Questionnaire completed	129(89.0%)	Questionnaire completed	123(87.9%)
No response	15(10.3%)	No response	11(7.9%)
eq5d reading	126(86.9%)	eq5d reading	121(86.4%)
iop reading	138(95.2%)	iop reading	124(88.6%)
Complete cost data	129 (89.0%)	Complete cost data	120 (85.7%)
withdrawal from study	1(0.7%)	withdrawal from study	5(3.6%)
deceased		deceased	1(0.7%)
**36 months**		**36 months**	
Questionnaire completed	134(92.4%)	Questionnaire completed	123(87.9%)

No response	7(4.8%)
eq5d reading	125(86.2%)
iop reading	130(89.7%)
Complete cost data	131 (90.3%)
withdrawal from study	2(1.4%)
deceased	2(1.4%)

No response	8(5.7%)
eq5d reading	116(82.9%)
iop reading	124(88.6%)
Complete cost data	122 (87.1%)
withdrawal from study	6(4.3%)
deceased	3(2.1%)

**Across all time points**	
Complete cost data	105 (72.4%)
Complete QALY data	96 (66.2%)
Complete cost and complete QALY data*	93 (64.1%)

**Across all time points**	
Complete cost data	100 (71.4%)
Complete QALY data	88 (62.9%)
Complete cost and complete QALY data*	86 (61.4%)

* Data used for complete case cost-effectiveness analysis

Supporting Table 1 Parametric time-to-event Weibull regression and Logistic regression results

Parametric time-to-event Weibull regression results for estimating probability of glaucoma surgery					
**Variable**	**Hazard Ratio**	**Std. Error**	**P-value**	**[95% CI**	
Treatment (LE)	0.050	0.037	0.000	0.012	0.209
Mild	2.248	1.836	0.321	0.454	11.142
Moderate	9.028	6.683	0.003	2.116	38.516
Severe	7.853	6.211	0.009	1.666	37.006
_cons	0.000	0.000	0.000	0.000	0.001
p	0.871	0.137	-	0.640	1.185
**Logistic regression results for estimating probability of progression**					
**Variable**	**coefficient**	**Std. Error**	**P-value**	**[95% CI**	
Treatment	-0.360	0.368	0.328	-1.082	0.361
Mild	16.759	0.378	0.000	16.018	17.499
Moderate	15.447	0.490	0.000	14.487	16.407
Severe	15.424	0.418	0.000	14.605	16.242
_cons	-17.083	0.368	0.000	-17.804	-16.362

Supporting Table 2 Health service utilisation by treatment allocation group

Variables	Randomized to early lens extraction		Randomized to standard care	
**All patients**	(*N* = 208)		(*N* = 211)	
**Patients in the UK**	(*N* = 145)		(*N*= 140)	
**Treatment received in the study eye**	**N**	**n (%)**	**N**	**n (%)**
Lens extraction	145	143 (98.62)	140	0 (0)
Standard care	145	1 (0.68)	140	139 (99.28)
No recorded treatment	145	1 (0.68)	140	1 (0.72)
**Treatment received in the other eligible eye**				
Lens extraction	145	42 (28.97)	140	0 (0)
Standard care	145	1 (0.68)	140	61 (43.57)
No recorded treatment	145	21 (14.48)	140	3 (2.14)
**Health Care Utilization**				
**Subsequent procedures in the study eye**				
Lens capsulotomy*	123	9 (7.32)	113	0 (0)
Iridoplasty	122	0 (0)	113	10 (7.96)
Iridotomy	123	2 (1.63)	117	10 (8.55)
Trabeculectomy	122	1 (0.82)	114	5 (4.39)
Cataract surgery	122	0 (0)	116	20 (17.24)
**Subsequent procedures in the other eligible eye**				
Lens capsulotomy	123	6 (4.88)	113	0 (0)
Iridoplasty	122	0 (0)	113	6 (4.42)
Iridotomy	124	1 (0.81)	117	6 (5.13)
Trabeculectomy	122	1 (0.82)	114	1 (0.88)
Cataract surgery	126	6 (4.76)	115	6 (5.22)
**Subsequent procedures in the non-eligible eye**				
Lens capsulotomy	123	3 (2.44)	113	0 (0)
Iridoplasty	122	0 (0)	114	3(2.63)
Iridotomy	124	13(10.48)	125	75 (56)
Trabeculectomy	122	0 (0)	113	0 (0)
Cataract surgery / lens extraction	127	56 (44.09)	117	5 (4.27)
**Medication in the study eye**				
Prostaglandin	128	42 (32.81)	131	101 (77.10)
Beta blockers	123	18 (14.63)	122	46 (37.70)
Carbonic anhydrase inhibitor	122	12 (9.84)	120	37 (30.83)
Pilocarpine	123	1 (0.81)	114	17 (14.91)
Alpha agonists	122	3 (2.46)	115	12 (10.43)
**Medication in the other eligible eye**				
Prostaglandin	128	19 (14.84)	131	48 (36.64)
Beta blockers	123	4 (3.25)	122	19 (15.57)
Carbonic anhydrase inhibitor	122	6 (4.92)	120	16 (13.33)
Pilocarpine	124	3 (2.42)	114	7 (6.14)
Alpha agonists	122	1 (0.82)	115	5 (4.35)
**Medication in the non-eligible eye**				
Prostaglandin	128	22 (17.60)	131	43 (33.86)
Beta blockers	122	12 (9.84)	122	19 (15.70)
Carbonic anhydrase inhibitor	120	7 (5.74)	120	13 (10.83)
Pilocarpine	123	5 (4.07)	114	10 (8.77)
Alpha agonists	122	2 (1.64)	115	5 (4.35)
**Primary health care utilization**		**Mean (SD)**		**Mean (SD)**
GP visit	108	1.68 (2.74)	103	2.24 (4.32)
GP call	108	0.39 (1.40)	103	0.45 (1.48)
GP home visit	108	0.01 (0.09)	103	0.08 (0.69)
Practice nurse consultation	108	2.32 (5.24)	103	2.73 (3.66)
District nurse consultation	108	0.18 (1.19)	103	0.7 (3.02)
Community optician/optometrist visit	108	1.88 (1.80)	103	1.95 (1.89)
**Secondary care**				
Ophthalmologist visit	128	4.86 (5.30)	123	5.05 (4.67)

*Some procedures have been used more than one time

Supporting Table 3 Results of model based deterministic sensitivity analysis for different time horizons

**Scenario**	**Cost (£)**	**Incremental cost (£)**	**QALYs**	**Incremental QALYs**	**Incremental cost per QALY (£)**	**Probability of being cost-effective at Rc £20 000**
**3-years' time horizon**						
Scenario 1: Excluding costs of procedures and medications in non-eligible eyes from total cost estimates						
Standard care	1,435	–	2.485	–	–	–
Lens extraction	2,153	718	2.546	0.061	11,721	85%
Scenario 2: Cost and utility inputs based on full multiple imputation analysis						
Standard care	1,583	–	2.428	–	–	–
Lens extraction	2,385	802	2.525	0.097	8,267	97%
Scenario 3:Inclouding indirect and patient's cost in total cost estimates						
Standard care	2,641	–	2.484	–	–	–
Lens extraction	3,681	1,040	2.546	0.0620	16,772	63%
Scenario 4: Using alternative regression to estimate mean cost and utility (GLM)						
Standard care	1,560	–	2.472	–	–	–
Lens extraction	2,522	962	2.541	0.069	13,952	76%
Scenario 5: Changing the mean age of the cohort from 67 to 50 years old						
Standard care	1,567	–	2.527	–	–	–
Lens extraction	2,486	919	2.591	0.064	14,357	73%
Scenario 6: Estimating time to glaucoma and cataract surgery using parametric survival model using exponential distribution						
Standard care	1,545	–	2.484	–	–	–
Lens extraction	2,505	960	2.547	0.063	15,255	70%
**5-years' time horizon**						
Scenario 1: Excluding costs of procedures and medications in non-eligible eyes from total cost estimates						
Standard care	2,154	–	3.903	–	–	–
Lens extraction	2,571	417	3.982	0.079	5,292	95%
Scenario 2: Cost and utility inputs based on full multiple imputation analysis						
Standard care	2,286	–	3.824	–	–	–
Lens extraction	2,661	375	3.962	0.139	2,708	100%
Scenario 3:Inclouding indirect and patient's cost in total cost estimates						
Standard care	3,987	–	3.903	–	–	–
Lens extraction	4,368	381	3.981	0.078	4,864	95%
Scenario 4: Using alternative regression to estimate mean cost and utility (GLM)						
Standard care	2,282	–	3.896	–	–	–
Lens extraction	2,827	545	3.978	0.082	6,674	93%
Scenario 5: Changing the mean age of the cohort from 67 to 50 years old						
Standard care	2,345	–	4.031	–	–	–
Lens extraction	2,816	471	4.112	0.082	5,764	92%
Scenario 6: Estimating time to glaucoma and cataract surgery using parametric survival model using exponential distribution						
Standard care	2,271	–	3.903	–	–	–
Lens extraction	2,792	520	3.982	0.079	6,557	92%
**10-years' time horizon**						
Scenario 1: Excluding costs of procedures and medications in non-eligible eyes from total cost estimates						
Standard care	3,360	–	6.617	–	–	–
Lens extraction	3,344	-16	6.726	0.110	dominate	96%
Scenario 2: Cost and utility inputs based on full multiple imputation analysis						
Standard care	3,489	–	6.508	–	–	–

Lens extraction	3,210	-279	6.712	0.205	dominate	100%
Scenario 3:Inclouding indirect and patient's cost in total cost estimates						
Standard care	6,288	–	6.623	–	–	–
Lens extraction	5,695	-593	6.727	0.105	dominate	98%
Scenario 4: Using alternative regression to estimate mean cost and utility (GLM)						
Standard care	3,489	–	6.620	–	–	–
Lens extraction	3,408	-81	6.725	0.105	dominate	96%
Scenario 5: Changing the mean age of the cohort from 67 to 50 years old						
Standard care	3,754	–	7.287	–	–	–
Lens extraction	3,482	-272	7.399	0.113	dominate	95%
Scenario 6: Estimating time to glaucoma and cataract surgery using parametric survival model using exponential distribution						
Standard care	3,493	–	6.620	–	–	–
Lens extraction	3,363	-130	6.728	0.107	dominate	96%
